# Non-Haredi Arts Therapists’ Perceptions of Therapy With Ultra-Orthodox Children

**DOI:** 10.3389/fpsyg.2021.599872

**Published:** 2021-02-04

**Authors:** Lali Keidar, Dafna Regev, Sharon Snir

**Affiliations:** ^1^Graduate School of Creative Arts Therapies, University of Haifa, Haifa, Israel; ^2^Department of Art Therapy, Tel-Hai College, Tel Hai, Israel

**Keywords:** arts therapy, ultra-Orthodox Jews, children, cross cultural, therapists perceptions

## Abstract

Studies have underscored the complexity of the encounter between ultra-Orthodox (Haredi) society and psychotherapy, as well as the challenges involved in developing a therapeutic relationship in cross-cultural therapy. However, there is scant research on therapy for ultra-Orthodox children, especially when it comes to arts therapies that take place in a cross-cultural setting. The current study examined the perceptions of 17 arts therapists (including visual art therapists, dance/movement therapists, psychodramatists, music therapists and bibliotherapists) who are not ultra-Orthodox, and who currently work or have previously worked with ultra-Orthodox children. Semi-structured interviews were conducted with the therapists and analyzed using the principles of Consensual Qualitative Research. The study covered four domains: (1) perceptions of the significance and objectives of arts therapy with ultra-Orthodox children; (2) the influence of the cultural difference between therapist and client on the emotional experience and the therapeutic relationship; (3) the use of arts in therapy; (4) systemic aspects. The findings indicate significant perceptual and value-based disparities between therapists and clients, which pose difficulties and challenges to all participating parties and require therapists to be highly sensitive. Aside from the difficulties, the findings suggest that this cultural difference may also have certain advantages for clients as well as therapists. The findings likewise attest to the multifaceted process of change that is taking place within Haredi society in its attitude toward psychotherapy in general and arts therapy in particular.

## Introduction

Haredi Jews are a distinct segment within Jewish society in Israel and in the world, representing the most ultra-Orthodox factions of Judaism. As of 2019, the Haredi sector in Israel constituted 12% of the country’s population (Cahaner and Malach, 2019), and continues to grow due to its high birth rate and low assimilation into the general population. Haredis are concentrated primarily in areas in New York and New Jersey, and in the cities of Bnei Brak and Jerusalem in Israel (Greenberg and Witztum, 2013). Haredi society is extremely traditionalist, collectivist, and patriarchal, conducting itself as a closed community with emphasis on faith in God, strict obedience to Jewish law, and staunch loyalty to the community (Freund and Band-Winterstein, 2017; Nadan and Ganz, 2018). Haredi society does not form a homogeneous group. It consists of the three main factions of Hasidim, Lita’im (Lithuanians) and Sephardic Haredim, which also branch out into subgroups that differ from each other in many ways including with respect to customs, leadership, educational institutions and attire (Zicherman and Cahaner, 2012). Nonetheless, a clear system of values can be considered common to all factions in Haredi society, including adherence to religious norms and rabbinic decrees, alongside resistance to a secular lifestyle (Freund and Band-Winterstein, 2017). Haredi Jews are distinguished by their dress, which emphasizes modesty, and their restrictions on inter-gender mixing from an early age (Greenberg and Witztum, 2013). The poverty rate in Haredi society is very high, due to the Haredi household structure, which is characterized by large families and a low rate of men in the labor force (Cahaner and Malach, 2019). Despite the significant gaps between Haredi and non-Haredi society, which are manifested mainly in terms of exemptions from military service and restricted use of computers and the Internet, there is a noticeable trend toward greater integration of Haredim into Israeli society in general, including into higher education and the job market (Cahaner and Malach, 2019). The present research examines the perceptions of non-Haredi arts therapists regarding work with children from the Haredi community. This kind of therapy is defined as cross-cultural therapy, a term that points to the significant influence that the cultural difference between the different parties has on the process (Fung and Lo, 2017). The client’s system of cultural beliefs affects their self-perception, their expectations from relationships, their willingness to expose their inner self, as well as their perception of the treatment and its objectives (Cross and Bloomer, 2010; Fung and Lo, 2017). Clients from different cultures may have different ways of expressing distress, or even different ways of understanding the source of their distress and how it might be alleviated (Fung and Lo, 2017). Likewise, cultural beliefs regarding the subject of mental illness and health sometimes lead to the development of a stigma, which presents an obstacle and amplifies the feelings of shame attached to experiencing mental distress and seeking treatment (Cross and Bloomer, 2010; Gopalkrishnan, 2018). A client’s traditionalist-collectivist cultural background may present significant challenges in the course of therapy, since the Western system of cultural values underlying the practice of psychotherapy is fundamentally different from the traditionalist system of cultural values (Qureshi and Collazos, 2011).

Studies examining psychotherapy in Haredi society show that despite the progress in terms of openness to psychotherapy and the legitimacy of receiving aid from outside the Haredi world (Freund and Band-Winterstein, 2013), there is still noticeable ambivalence, suspicion and hostility toward external sources of mental health support (Popovsky, 2010; Freund and Band-Winterstein, 2017). These attitudes are sometimes reflected in a restricted ability to cope with doubts, a hard time engaging in introspection (Hess and Pitariu, 2011), and an expectation that the client will be provided with practical recommendations (Hess, 2018). Furthermore, the fact that the community occupies such a central position in the life of the individual may undermine the very legitimacy of focusing on one’s individual identity (Freund and Band-Winterstein, 2017; Schlesinger and Russo-Netzer, 2017). It may also lead a client to be unwilling to expose hardships or cooperate during therapy due to fears of harming their own or their family’s social status within the community (Greenberg and Witztum, 2013; Barth and Ben-Ari, 2014). On the other hand, other studies suggest that the therapist’s provenance from outside the community may increase the client’s sense of trust and engagement due to the decreased possibility of exposure within the community (Stolovy et al., 2012; Freund and Band-Winterstein, 2013). In the case of child psychotherapy, the parents may experience significant distress stemming from their sense of responsibility for the child’s spiritual and religious upbringing (Schnitzer et al., 2011).

The complexity involved in the Haredi individual’s encounter with therapy may be especially high when it comes to arts therapy. Beyond the fact that there are multiple Halachic ordinances restricting the practice of various art forms, the Haredi community tends to perceive the arts as a means of cultural, spiritual and moral education; the possibility of using the arts as a language to express one’s inner world, on the other hand, remains fairly limited (Sperber, 2010; Sari, 2013).

Nevertheless, arts therapy exists in the Haredi community, and has in fact been increasing in popularity in recent years. Research on the subject, however, is still extremely scant and consists exclusively of studies that focus on visual art therapy (Padolski-Kroper and Goldner, 2020) and dance and movement therapy (Suskin and Karnieli, 2015). These studies indicate that Haredi society views arts therapy as a less legitimate form of therapy, because they consider it less practical or constructive. Moreover, this type of therapy causes clients to face their own difficulty with playfulness and creative expression, in addition to a sense of anxiety about unrestrained expression. At the same time, it would seem that incorporating arts in therapy makes it possible to bypass the clients’ defense mechanisms and allows them to express emotions through distancing (Padolski-Kroper and Goldner, 2020).

In light of the paucity of research on the subject, the current study sought to conceptualize the therapeutic act in cross-cultural arts therapy for Haredi children by examining the experiences and perceptions of arts therapists from outside the Haredi community. The research questions were as follows: (a) How do non-Haredi arts therapists experience art therapies with ultra-Orthodox children? (b) What defines the specificity of the therapeutic relationship, if any, when the encounter is inter-cultural?

## Materials and Methods

### Participants

The sample consisted of 17 arts therapists between the ages of 31–62 (*M* = 43.52), who are presently working or have previously worked with children from the Haredi community. In addition to experience in treating children from the Haredi community, all the therapists had experience in arts therapies with secular children. All the therapists were non-Haredi women, most of them secular with the exception of three participants who identify as Religious Zionist (i.e., they belong to Modern Orthodox Jewish faction that espouses Zionist ideology). Their areas of specialization corresponded to those defined by the Israeli Association for Creative Arts Therapies (YAHAT). According to this official definition, arts therapies implement artistic creation and expression processes through one of six specializations (Orkibi, 2019): dance/movement (six therapists), bibliotherapy (four therapists), visual arts (three therapists), psychodrama (two therapists), and music (two therapists). One drama therapist dropped out of the study. The children treated by these therapists primarily had social difficulties, behavioral problems, attention deficit disorders, learning difficulties, and difficulties related to trauma and sexual abuse. The clients belonged to all factions of Haredi society; i.e., Hasidim, Lita’im, and Sephardic Haredim. The demographic data pertaining to the participants are detailed in Supplementary Table 1. The information is presented in purposefully broad terms in order to impede the participants’ identification.

### Procedure

Participants were recruited by means of the snowball sampling method. Initially, the first author contacted therapists who had worked at Arugot – a therapy facility designated to treating members of the Haredi community in the North of Israel. This therapy facility employs secular therapists, but its policies and atmosphere are compatible with the principles of Haredi society. For example, the administrative staff are Haredi, and the therapists are required to be dressed adapted to the ultra-Orthodox dress code. Parents pay for therapy at Arugot but are reimbursed by their health maintenance organizations. Nine of the therapists who worked at Arugot agreed to be interviewed for this study. In the second phase of recruitment, several of the participants put the first author in touch with other therapists who had worked at Arugot in the past. The researcher contacted these therapists and four of them agreed to join the study. Later the first author contacted four more therapists working in other regions in Israel. All interviews took place between October 2019 and March 2020. Each interview lasted approximately 1 h. Eleven of the interviews were conducted face-to-face, and the remaining six were conducted over the phone. With the participants’ consent, the interviews were recorded and transcribed, and once all the participants had been interviewed, the transcriptions underwent data analysis and processing.

### Data Collection

Semi-structured interviews were conducted with the participants by the first author who is likewise an art therapist. The author had no previous personal or professional interactions with any of the study’s participants. The author utilized an interview guideline consisting of open-ended questions to ask the therapists about their general perceptions regarding the practice of arts therapy with Haredi children. The interviews probed three main topics: (1) Psychotherapy in Haredi society: the specificity of therapeutic work with Haredi clients, the effects of Haredi culture on therapy (e.g., “Does the fact that your clients are from the Haredi community have an effect on therapy?”), therapy goals, key dilemmas, content (e.g., “Can you identify content that comes up in therapy as opposed to content that does not come up?”), and the relationship with the client and the child’s parents; (2) arts therapies in Haredi society: The place and roles of the arts in therapy, the specificity of the use of the arts in therapy with Haredi children, therapeutic qualities of the arts (e.g., “Arts therapy can evoke exposure, emotional expression, playfulness and release. Did you experience these features in therapy for Haredi children and if so, how?”); (3) the cross-cultural encounter: the effects of the cultural difference between the therapist and the client on the therapy and the therapeutic relationship, advantages, and disadvantages of cultural differences. In most cases the questions were formulated as they were worded in the interview protocol. However, since the interviews were semi-structured, slight changes were sometimes made in the wording of the questions and follow-up questions were asked when needed to delve into the content of the interviews. As part of the effort to streamline the process, the interviewer used probes such as asking the therapists to give examples and repeating the participants’ answers in her own words.

### Data Analysis

The present study was conducted in accordance with procedures defined Consensual Qualitative Research (CQR) (Hill et al., 1997, 2005; Hill, 2015), a rigorous qualitative approach that allows for an in-depth exploration of individual experiences. Research according to the CQR approach strives to develop detailed descriptions of processes and perceptions through a data analysis consisting of three central steps (domains, core ideas, and cross-analyses) that are carried out by several judges and then reviewed until a consensus is reached (Hill et al., 2005; Hill, 2015). The CQR is considered well suited for the study of complex phenomena such as therapeutic processes (Hill et al., 1997), and therefore is frequently used in counseling and psychotherapy studies (e.g., Coutinho et al., 2011; Daniels et al., 2015; Caperton et al., 2020). It is particularly useful for examining topics that have not yet been explored or for a practice for which there are few theoretical foundations (Hill et al., 1997). For these reasons, it is used in fields that are relatively new in academic research, such as arts therapies (e.g., Belity et al., 2017; Harpazi et al., 2020).

In accordance with the principles of the CQR, in the first stage, three interviews were analyzed separately by each member of the research team, all of whom are art therapists themselves, with the aim of identifying the central domains emerging from the data for each of the interviews separately. At the end of this stage of analysis the researchers came together to compare their findings and reach a consensus concerning the definition of the domains for the three interviews (cross analysis). Following that, the first author analyzed the rest of the interviews while categorizing the data according to the consensually agreed-upon domains and adjusting as necessary based on the new materials. In the next stage of analysis, the research team went over the materials that had been categorized under each domain separately, defined core ideas for each domain and then assembled to discuss in order to reach a consensus.

The prevalence of the core ideas is defined on a three-tier scale: the term “most therapists” is used to describe a phenomenon that was identified in over 75% of all interviews, the term “some therapists” describes a prevalence of 25–75%, and the term “a few therapists” describes a prevalence of less than 25% of cases (Hill, 2015).

### Professional Ethics and Confidentiality

Both the researcher contacting the therapists and the consent form they were asked to sign explicitly stated that the therapists were not obligated to participate in the study and that they were free to withdraw from it at any stage. They were promised that their identity would remain confidential throughout the study and the publication process. There was particular emphasis placed on protecting the privacy of the clients, and the therapists were instructed to give only general and anonymous examples. Any identifying details were redacted in the interview transcription stage and the recordings were destroyed at the end of the transcription process. The present study was approved by the ethics committee for the Faculty of Social Welfare and Health Sciences at the University of Haifa.

## Results

### Perceptions of the Significance and Objectives in Arts Therapy With Ultra-Orthodox Children

#### The Overall Perception of Therapists Treating Haredi Children Is That It Is No Different From Treating Children From Outside Haredi Society

Despite the specificities of this population, most therapists stressed that administering arts therapy to ultra-Orthodox children is in large part similar to administering arts therapy to children in general. Thus the therapists sought to emphasize that the fact that the clients were children was more important and central than the culture in which they were brought up – As stated by some of the therapists: “They’re just children like any other.”

#### The Therapists Did Identify Unique Objectives in Treating Haredi Children Which Are Influenced by Cultural Factors

Notwithstanding the statements above, most therapists identified a few objectives which they perceived as specific to administering arts therapy to Haredi children. The therapists made this distinction based on their experience in treating secular children. One main objective which emerged from some of the interviews focused on bolstering the child’s self-expression. This objective revolves around developing the ability to express the self, including encouraging flexibility and broadening the child’s range of reactions to include ones that do not stem from cultural norms or ones that may even oppose them, such as refusal: “to allow another kind of expression, to allow me to connect to what it is I’m feeling.” Some of the therapists expounded that children from ultra-Orthodox society often find it difficult to be authentic, to express thoughts and feelings as individuals in the context of strict cultural norms: “It is very hard for them to talk about the self… they can talk about themselves in the space of their school, about what happened to them, but not about how they felt or what they thought.”

Another objective that came up in the statements of some therapists touched on the need to develop and evolve the children’s emotional vocabulary. According to these therapists, in many of these children’s households, especially ones that belong to certain ultra-Orthodox factions, the language of emotion is either impoverished or lacking entirely: “You can feel that there is no validation at home of the experiences the child is going through… the overall impression is that the whole mode of conduct at home is very technical.” As a result, Haredi children often display difficulties in recognizing and expressing emotions, especially complex emotions such as jealousy. The children, especially ones of primary school age, tend to express themselves through their behavior rather than talking about their feelings, a blockage that manifests itself in behavioral problems: “When there is no emotional vocabulary and it’s hard for me to express and say how I feel, that’s where the problem starts. The child acts out the thing rather than speaking the thing.”

Some therapists referenced growing up in a large family as a dominant cultural attribute which affects other therapeutic objectives. Thus, for example, one objective linked to the fact of growing up with multiple siblings is dealing with a sense of inability to handle the responsibility of taking care of younger brothers and sisters. Another objective is dealing with problems of wetting or soiling themselves as a result of the social acceptance of late toilet training, which possibly stems from the parents’ inability to toilet train when there is a large number of children in the house and they are preoccupied with household maintenance tasks. These therapists underscored the significance of therapy in this context as an opportunity to provide the child with exclusive and undivided attention: “to give them a warm place where the child can feel themselves again, which is hard when there are so many children at home.”

A few of the therapists shared their perception of bolstering the child’s ability to make choices as being an important therapy objective. These therapists suggested that the difficulties these children experience with choosing are likewise due to the realities of living in large families from a low socio-economic background: “They might say – I’ll take whatever there is… Because a lot of the time there is nothing, or they have to share with a lot of other children.” Moreover, a few therapists insisted that children in Haredi society have a strong need to please which is most likely developed by their upbringing: “The desire to please is there all the time. That’s how they are raised.”

A few of the therapists also mentioned working on anxieties in general and on Obsessive-Compulsive Disorder (OCD) in particular as an additional objective. These therapists claimed that the anxieties have to do with the feeling of inability to uphold the strict precepts of religious law: “They have very severe anxieties around the whole subject of upholding the Halachot [Jewish laws] and the Mitzvot.”

#### Perceptions of Arts Therapy and Its Significance in the Eyes of Haredi Society

When the therapists were asked to describe the manner in which, in their opinion, arts therapy is perceived in Haredi society by parents, educational staff and rabbis, their responses yielded quite a few contradictions and disparities. Some of the therapists agreed that there has been a vast improvement over recent years in terms of the willingness and desire to participate in therapy, both in the context of educational frameworks, and in private clinics and institutes. These therapists described a certain degree of respect accorded to the therapist, who is perceived as a professional, and mentioned that rabbis too seem to have a greater understanding of the importance of therapy, and their perception has an influence on the disposition of the Haredi population as a whole: “today there is a kind of understanding that therapy is not just for crazy people, there is an understanding that, especially for children, it does them good.” Nevertheless, a few of the therapists argued that the stigma of receiving therapy is still widespread, since it attests to a deficiency within the person or the family: “it is a great shame to go to therapy, it means that there’s something wrong with you.” These therapists also implied that even when there is a certain willingness to receive therapy, there is still a staunch objection to psychiatric diagnosis.

A similar inconsistency arose in relation to the Haredi population’s understanding of the meaning of therapy, with a few of the therapists claiming that despite a willingness to receive treatment, those seeking it do not have a good understanding of what therapy entails. Some therapists, on the contrary, opined that today there is a greater understanding of the meaning of therapy, and that some clients even have clear objectives and requests pertaining to coping tools when they come in for their first session. These therapists claim that sometimes parents bring their children to therapy with the expectation that it will solve all of the child’s and the family’s problems: “just straighten out some of the kinks in their kid for them… they want you to fix their child.” According to some therapists, the parents demand quick solutions for the therapy objectives that drove them to send their child to therapy, and if the desired results fail to be obtained, they seek to understand the kind of work that has been done in the course of therapy. These therapists were divided among themselves with regards to the ability of parents to perceive therapy as a process. However, most therapists expressed encountering difficulties in this respect: “there is a great desire for some kind of recipe that would explain how things are or what’s working, and less of a willingness to understand that it’s a process.”

In describing the perceptions of the meaning of therapy among Haredi educational staff, once again opinions were conflicted. Thus, alongside the descriptions by a few therapists about narrow and dogmatic outlooks that focused on the direct benefits of therapy, there were also a few therapists who reported a great deal of appreciation accorded to the therapists’ work.

#### Therapy Referral Agents and Reasons for Referral in the Eyes of Haredi Society

A few of the therapists underlined the fact that most parents do not seek therapy for their children of their own initiative, but rather following the suggestion of educational staff – school and kindergarten teachers, or rabbis.

The main reason for referral which came up in some therapists’ statements was social problems, such as introversion and loneliness. There are even cases where the parents insist on focusing on social problems despite the background presence of far more complex issues such as severe sexual abuse. It is manifest that the parents find it very important for their children to fit in well in society, which a few therapists consider largely the result of the community being central to the life of the Haredi individual: “the issue of being really well adjusted socially is very important for them… the importance accorded to how you are perceived is very much magnified.”

Another principal cause of referral mentioned by some therapists had to do with learning deficiencies, attention deficiencies, and difficulties with organization, which are often accompanied by outbursts of anger, violence and difficulties regulating emotions. Some therapists described the great deal of pressure parents place on scholastic achievements, and added that oftentimes parents turn to therapy following displays of behavior that they perceive as abnormal and disruptive to the child’s studies: “the idea that the child has to behave properly when it comes to school… it is much more stressful for the family.” Nevertheless, a few of the therapists described parents who focus on the child’s emotional wellbeing even in children with attention disorders, and seek to bolster the child’s confidence: “I have a few kids with ADHD, but the parents bring them to therapy because they want to raise their confidence.”

One cause of referral particular to this population, which was mentioned by a few of the therapists, was behaviors that were incompatible with the rules and norms of Haredi society, such as modest dress, respecting one’s parents and elders or upholding the Halacha: “They want good kids who do as they should.”

Most therapists claimed to often notice disparities between the therapy objectives as they perceive them as opposed to the perception of the parents. However, it would seem that in most cases, the therapists place the parents’ perceived objectives at the center of their work, and either hold off on working on their own perceived objectives or work on them indirectly, while maintaining a realistic perspective on the clients’ real lives to which they return at the end of the therapy session: “I can’t open up big gaps because at the end of the day they go back to their parents… it would be irresponsible of me.”

### Influence of the Cultural Difference Between Therapist and Clients on the Emotional Experience and the Therapeutic Relationship

#### Cultural Disparity as an Opening for Candor Including Revelation of Secrets

Most therapists claimed that their not belonging to Haredi society influenced the willingness of the children and the parents to reveal things about themselves. Some therapists talked about parents and children sharing experiences and dreams during therapy sessions which include content that is forbidden or frowned upon in Haredi society. These therapists explained that the revelation of such content can take place due to the fact that the therapist does not belong to the ultra-Orthodox community and thus there is little risk of her exposing the secret revealed to her: “I’m not part of the community, and therefore the fact that I am holding on to their secret can’t lead it to be exposed, which might influence all kinds of things that have to do with their wellbeing.” Some therapists added that clients are also able to be candid because of the therapist’s lack of judgment toward the revealed content, since she does not espouse the ethical standards and the value system of Haredi society: “I came with a slightly more relaxed and less rigid attitude into this set of rules and regulations of the Haredi world.” As a result, the clients and their parents are more willing to share and reveal even very traumatic experiences, such as multi-generational sexual abuse. A few of the therapists suggested that sharing takes place out of a perception of the therapist as a window of opportunity to experiment with fantasies and forbidden wishes: “I live their dream. I allow them this window of being without judgment.”

Even so, it is evident that the fear of exposure is very much present during the therapy sessions. Even though a few of the therapists claimed that there is less secrecy and aversion to revealing complex content than before, most therapists shared having the sensation that there were things being kept from them: “There is a feeling of secrecy.” Thus, for instance, the children tend not to share much about their family and the dynamics at home, and the parents sometimes avoid sharing such vital content as sexual assault or psychiatric diagnosis which the therapist only discovers during therapy. The reason for this, according to a few therapists, may have to do with the Halachic ordinance against defamatory speech, which prevents the clients and their parents from speaking about the bad deeds committed by others, and the claim was put forth that some use religion as an excuse for concealing the truth. Furthermore, a few of the therapists mentioned that in some more complicated cases, such as ones involving sexual assault or fear of the client leaving the religion, the community put an end to the therapy and the client was transferred for therapy by a therapist within the Haredi community.

#### Cultural Differences Lead to Missed Nuances and Difficulties in the Therapist’s Ability to Understand

Some therapists revealed that significant language barriers exist in arts therapy with ultra-Orthodox children. Beyond the obvious linguistic obstacles that arise when the clients speak a different language – namely, Yiddish, spoken in many Haredi households – language barriers appear even in treatments where the client and therapist speak the same language (Hebrew). Some therapists expounded that barriers can arise following their own lack of familiarity with the meaning of certain expressions, symbols, sayings, codes and rules, as well as disparities in body language: “We just don’t speak the same language. A lot of it is in the subtext, in the ability to decipher both the hidden messages and the body language.” These therapists’ statements indicate that these disparities and barriers have an effect on the therapy and the therapeutic relationship. Thus, for example, some therapists described cases where they acted based on their own conceptual world, a world that is often in conflict or mismatched with the conceptual world of the client, which led to misinterpretations, created emotional distance, and even unintentionally caused the client distress. In some cases, the cultural disparity created a veritable disconnect and led to the cessation of therapy. It would appear then that language barriers sometimes prevent the possibility of professional discourse: “Our lack of knowledge silences us… it’s very hard to stand up as a professional and explain… it’s as if they bring a different kind of knowledge which belittles us.” A few of the therapists, on the other hand, opined that missing out on nuances may also serve as an advantage, since it forces the therapists to be more intuitive in their work.

#### The Therapists Try to Sensitively Adapt Themselves to the Clients’ Culture

As shared by some therapists, the linguistic disparities force them to adapt their language to that of their interlocutors: “I feel like I choose my words very carefully… Beyond the processes of introspection, of looking at what’s going on, I also adapt my language to theirs.” Some therapists explained that they use expressions taken from the religious world in order to describe and mediate therapeutic terms, and that they likewise adjust their language to resemble the Haredi way of speaking, which is more proper and less casual. This way of speaking forgoes the use of slang, on the one hand, but on the other hand, it also sometimes lacks words and expressions of a higher register. Moreover, the use of certain words is forbidden or frowned upon, such as words describing bodily functions and secretions, and the therapists must adapt their speech accordingly.

Some therapists said that in order to adapt themselves they mostly consult with the parents, and sometimes with the children themselves, with the rabbis of the community, or with other therapists. In some cases, they have recourse to supervision. Another focal point of adaptation mentioned by some therapists is the therapeutic content. Some therapists explained that they frequently check what is allowed and what is forbidden in Haredi society, and consult with parents about any specific boundaries which the therapist mustn’t overstep, or if there is any specific content that they do not wish to be included in the therapy sessions. Likewise, they are careful about revealing content from their own personal lives, out of the assumption that exposure to content that does not fit in with the client’s worldview may have an adverse effect on the therapy: “There is more attention paid to the details of what I say and what I do…and how much I share.” Furthermore, some therapists explained that they must exercise caution about applying their own set of values in their approach to the client, since this may provoke internal conflict in the client and cause them further distress: “This automatic approach that I come from, of encouraging her to rebel and to kick off this oppression she lived through, it was very problematic and could have gotten her in trouble.” Nevertheless, some therapists qualified this notion and stated that in some cases they mustn’t lose sight of their professionalism and that some things must be said even though they might go against the Haredi worldview.

Two additional areas in which the therapists have to adjust themselves include the need to adapt their clothing in order to comply with the rules of modesty customary in Haredi society (long skirts, long sleeves, closed shoes), as some of the therapists described, and the need to uphold the laws of Kashrut when it comes to food served in the context of therapy (in instances where the client and therapist cook together during treatment), which a few mentioned.

Some therapists also spoke of the need to respect the client and their worldview, even in instances when they feel the urge to disagree: “There are a lot of red lines, you have to know about them, you have to respect them when they differ from your own worldview or even when you disagree with them.” In addition, some therapists mentioned the importance of being familiar with the heterogeneity of Haredi society, and the need to distinguish between different factions in terms of what they allow and what they forbid: “There is so much variance that you have to learn to get to know who you are working with.” Some therapists emphasized the need to be humble and patient, and to be willing to constantly learn from the mistakes they make in the course of therapy.

#### The Therapists Experience Distress as a Result of the Cross-Cultural Encounter

Some of the therapists shared that they experience a measure of distress as a result of having to adapt themselves. Thus, for instance, the therapists revealed that the need to change the way they dress was sometimes experienced as an invasion of privacy, especially when the children asked questions about their dress or resorted to touch: “The questions the children asked me, why am I not wearing a wig or long socks, and they would even touch me physically, it embarrassed me and felt extremely invasive.” The discomfort described by some therapists was exacerbated by the feeling that they were meticulously scrutinized by the clients’ parents: “I may have imagined it, and I may have not, but they would also scan me with inquisitive glances.” Moreover, some therapists shared that having to make adaptations impedes their ability to act authentically, and increases their alertness and caution, which hampers their ability to treat the clients: “The main difficulty I experienced was the whole issue of being cautious, of not being able to be authentic.”

Alongside the aforementioned causes of distress, some therapists pointed out difficulties in dealing with their own critical outlook on different aspects of Haredi society. For instance, some of the therapists shared their criticisms of the high expectations placed on children in Haredi society, the great levels of responsibility and restrictions they have to deal with, which prevent them from being able to behave like children: “Everything has to be meticulous and organized, she has to act a certain way and she has to help her mother… and all those chores… she’s an old woman before her time.” Some therapists went on to speak about the distress they feel when dealing with young parents who have a large number of children even in cases where there are genetic problems present or difficulties in fulfilling parental functions. These therapists felt that these situations put the children at risk and hamper their emotional capacities. And yet, despite their intense feelings on the subject, A few of the therapists noted that they cannot discuss these issues with the parents and the criticism remains unspoken: “It’s a criticism, it’s a dilemma, and it’s like something you can’t touch on. There’s no discussion about bringing so many children into the world.”

Some therapists expressed feeling critical of the tendency to hide things in the Haredi community, and mentioned experiencing unease in situations where children had been charged with keeping a family secret. They especially noted situations where vital information is being kept from the child, such as their mother’s pregnancy, revealed only on the day she returns from the hospital with an infant. According to these therapists, such situations are distressful to the child who then has to adapt quickly and without support or mediation: “There is a very prominent tendency not to ask, or not to know in a sense… It’s an experience of a lack of control, which manifests itself later in very severe reactions on the part of the children.” Some therapists added that their discomfort increased when they are asked to endorse the family’s conduct and their policy of secrecy.

In addition, some therapists talked about difficulties dealing with their own criticism toward the general attributes of Haredi society, including political issues, displays of hierarchical thinking and racism: “It’s moments when you feel that most basic stigma in your heart. That point of thinking: you are not a just society.” A significant and dominant point of distress raised by a few of the therapists stemmed from disparities in nationalist ideology. These therapists described situations, which they found particularly distressful, wherein they felt a lack of respect and a disparagement of aspects related to their nationalist-Zionist values, an ideology toward which there is resistance in Haredi society. Thus, for example, the therapists described cases where clients’ parents would stare at them as they stood up to observe a moment of silence during the alarm sounded on Memorial Day, as well as instances of clashes between the clients and themselves around the subjects of Independence Day and mandatory military service. A few therapists explained that the anger and hostility they felt in these instances stemmed from the fact that they were not being treated with the esteem that their efforts to adapt themselves and to provide a sensitive and respectful therapy deserved: “We’re expected to respect the religion and all the rules… but something that is so significant to us… it was very problematic for me this whole subject.”

Another difficulty that emerged from some therapists’ statements touched on the way Haredi parents deal with any problem or issue they have with the therapist herself. Some therapists said that in many instances, parents addressed their complaints directly to the authority above the therapist (the management), without ever coming to her and discussing the matter with her. Such instances undermine the therapist’s status and often end up terminating the therapy: “They went straight to the principal and didn’t talk to me, they complained about me to the principal and so she got involved, with the best of intentions, but it did kind of demolish my authority.”

#### The Cross-Cultural Encounter Affects the Therapeutic Relationship

The findings show that the cultural disparity between therapists and ultra-Orthodox clients is significant and affects the therapeutic relationship. However, despite the prominent difference, a few therapists expressed finding themselves struggling with the dilemma of whether to address the difference directly or to repress it: “It’s a real issue, whether to lay it out on the table with the child, and it’s a dilemma. Whether my identity is something that should be discussed during therapy.” Some therapists said that the parents and the children know that they come from outside Haredi society, but some other therapists pointed out that the children are not fully aware of the cultural difference which is a source of confusion: “The kids over there find it really confusing: so they think of my hair as a wig, and let’s say if I get a haircut or something they’ll say ‘you’ve changed wigs!”.

Some therapists addressed positive effects of the cultural difference on ultra-Orthodox children’s participation in therapy. For example, the children feel the need to teach the therapist about Haredi society and by doing so they experience a feeling of control thanks to their ability to teach the other: “It gives the kids the feeling that they can teach someone something.”

Nevertheless, some therapists spoke of the obstacles the cultural difference places in the way of the developing therapeutic relationship. These therapists described a difficulty on the part of the clients to trust and bond with the therapist who is perceived as a foreigner. This is especially true for clients whose distress is underlain by an impaired ability to trust, such as victims of sexual assault: “She just couldn’t handle this kind of atmosphere, the way I’m talking to you right now… It was much too aggressive for her, too direct for her, very un-Haredi.” According to a few therapists, the cultural disparity oftentimes provokes a resistance to therapy on the part of the children and puts a strain on the relationship: “That’s the initial resistance… He says to me something like: you also want to lead me astray from my religion…what’s your value system compared to my value system.” The cultural difference also makes it harder for a few therapists to establish a therapeutic relationship, since they often feel disdain on the part of the clients in regard to their lifestyle. Some therapists expressed an overall feeling that they cannot be their natural selves, and that they need to be somewhat cautious about displaying any signs of closeness or intimacy. In order to bridge the cultural gap and allow the therapeutic relationship to flourish, a few therapists described looking for the common elements between the two worlds and emphasizing the transition from the outside world into the therapy room.

### The Use of Arts in Therapy

#### Arts Therapy Enables Clients to Broach Content That Cannot Be Verbally Discussed by Means of Distancing

Some therapists stated that the arts play a significant part in bringing up content from the child’s inner world, with emphasis on content that the child cannot talk about directly, such as everyday hardships, violence and sexual abuse: “The playing and the text and the creative work bring out the more difficult content… which, a lot of the time, it’s hard to talk about directly.” Likewise, through the arts, the children are able to explore and deal with their inner wishes and issues that are important to them. Some therapists expounded that art is a non-verbal medium for projection, which allows clients to broaden their experience and approach internal content indirectly, in a gentle manner: “That whole sophisticated mechanism of ‘I’m not talking about myself, I’m talking about this other thing.’ It goes a long way to breaking down their defense mechanisms.”

#### The Children’s Artistic Expressions Draw on Their Unique World of Content

Some of the therapists’ statements indicated that the unique world of content of children from the Haredi community is expressed in all the different kinds of arts therapy. Thus, for example, in visual art therapy, the children often draw stories, motifs and elements from the bible, such as the temple and the sacrificial altar. Moreover, drawings that depict subject matter from the children’s daily lives, such as drawings of their family, feature culturally marked choices in the character design and in the adherence to modesty codes, both in the way they are drawn and in the separation on the page between male and female characters. In psychodrama and bibliotherapy, the images and metaphors are drawn from the world of religion, the plot of the play or the story often includes events from the history of the Jewish people, and the characters the children portray are biblical characters, such as the High Priest. Music therapy often features music the children hear at home, including Sabbath chants and holiday songs. In dance/movement therapy, mundane items are infused with meaning that corresponds to the Haredi lifestyle: “A beanbag becomes a prayer book and a handkerchief always goes directly on the head.”

On the other hand, Some therapists sought to emphasize that despite the uniqueness of the children’s sphere of content, the arts allow them to express their emotional experience and inner world. For example, a recreation of the destruction of the Temple reflects a sense of failure and division in the family, whereas feelings of mental imbalance are often reflected in references to *yetzer hara* (the biblical term designating humanity’s inclination to do evil). In order to understand the emotional story behind the work, a few therapist resorted to phenomenological observation and discussions with the child about what the work means to them: “I try to understand together with the child… and the truth is that it opens up completely different things, things that have nothing to do with religion.”

#### Haredi Children’s Ability to Engage in Symbolic Play Is a Matter of Controversy

The therapists’ opinions were divided in regard to the ability of ultra-Orthodox children to engage with symbolism and play. Some therapists claimed that Haredi children are no different from any other children in their ability to play symbolic games, and that their main particularity in this respect is not related to the ability to play, but to the world of content expressed in the game: “I don’t think there’s a difference. It’s another world of content… the characters will look different, the story will utilize a different kind of language.” By contrast, some therapists described one challenge that arises in art therapies related to difficulties engaging in play among a large portion of Haredi children, which manifests in play that is mainly based on concrete references and in a limited capacity for imagination: “There are very well defined limits of the imagination there… the play tends to be very concrete sometimes.” It would appear that when the play, the story or the artwork draw on motifs from the world of Judaism, a world that is very familiar to the children, they find it especially hard to see past the rules and moral standards of Haredi society, and their imagination is blocked: “It was impossible to have a conversation with him that went beyond the moral of the story… He wasn’t at all open to something freer.”

#### The Otherness of the Therapeutic Space Allows for Different, Less Inhibited Behavior

Most therapists spoke about the difficulty that children from the Haredi community have letting loose, a difficulty which manifests itself during therapy in the children’s tendency to act in a restrained and stilted manner, a preference to sit at a table, occupying themselves with gathering up and arranging objects, and a focus on creating precise and aesthetically pleasing work. On the one hand, some therapists addressed the unease felt by the children faced with the uninhibited behavior of the therapist, as well as problems in experimenting with letting loose and a tendency to instead cling to rules and norms: “It scares them, they don’t like it… it’s very hard for them to be in that space.” On the other hand, according to some therapists, the children recognize therapy as a different space, apart from the one they are used to, where one can act more freely, and they react to it in different ways. Some therapists described the children’s’ excitement, which manifests itself in initial regressive reactions and in displays of enjoyment and curiosity: “They’re excited because I open the door for them to something they’re not really exposed to in their day-to-day lives.” Based on some therapists’ statements, we can surmise that the children’s reaction to the therapeutic space ranges between two extremes, with the first stage characterized by the children’s confusion and struggle to let go, which then leads them to behave in ways diametrically opposed to their normal behavior, running amuck and getting overexcited: “You can feel that sometimes it makes them go into overdrive mode, running wild.” For this reason, a few of the therapists have to vacillate between granting opportunities to cut loose and activities reflecting the need for restraint or balancing out the children’s reaction to liberation.

#### The Delicacy of Dealing With the Body and Sexuality During Therapy

Some of the therapists described various limitations in working with the body during therapy, such as the need to adjust, minimize and suppress their body movements to match the acceptable norms in Haredi society, which hampers their ability to be relaxed and natural. Some therapists added that the range of movement available to the children during therapy is also limited, due to the children’s tendency to play more spatially restricted games, such as table-top games, and their modest clothing which limits the potential for free motion: “She can’t roll over on the floor because she constantly has to hold her dress in place.” A few therapists also went on to describe the singular nature of the children’s movement, which involves less motion of the center of the body, with smaller and more restricted movements overall, and less freedom and flow. Moreover, some therapists explained that there are significant limitations in terms of language that references the body, to the point where the children have no words to describe various body parts, especially when it comes to private parts: “Between the belly and the legs, that whole part is missing. Which part of you touches the chair? The legs. What else? The back.” Accordingly, a few therapists find it hard to address the body in their work. They tend to avoid making movements that necessitate a discussion of the taboo body parts, and minimize work on content that has to do with the body, such as body image.

A few of the therapists reported being very much preoccupied with their distance from the clients and the possibility of touching them: “I am extremely aware of how far and how close I am. I can say that I am more careful about touch here than with other children.” The possibility of touch exists to a limited extent with children under the age of nine; it is more restricted with boys and it is absolutely forbidden with the fathers. A few therapists clarified that for the most part they cannot work with boys over the age of nine. Nonetheless, a few of the therapists claimed that the limitations on touch do not detract from the quality of the therapy or the ability to connect with the clients.

Some therapists recounted that contents related to sex and sexuality almost never arise during therapy sessions, even with adolescents. A few of the therapists underscored that when there is any mention of sexuality, they tend to see it as a red flag: “A child who broaches sexual content is a child who is going through something.” Moreover, a few of the therapists described ambivalence and apprehension on behalf of the parents in cases where they need to focus on this area, such as incidents of sexual assault or unusual preoccupation with sex on the part of the child: “The mother was really startled and was not willing to let me get into it.” Nevertheless, one of the therapists offered a contrary viewpoint on the subject and described a validation and openness toward sexuality among the Haredi community, based on their perception of sex as a mitzvah as opposed to a carnal urge.

#### The Therapist Makes Adjustments or Avoids Using Certain Artistic Tools Due to the Laws and Norms of Haredi Society

Most therapists stated that part of the adaptations they have to make for their Haredi clientele is centered around the artistic activities and involves making adjustments or even avoiding certain tools or artistic materials that may invoke content that is unacceptable in Haredi society. Thus, for instance, in working with text, a few therapists has to check whether the text is appropriate and get approval to use it. A few therapists emphasized that they cannot use books that feature impure (non-kosher) animals, or fantasy elements such as fairies, witches or dragons, and that sometimes they resort to changing the story’s content and replacing certain elements with names and concepts familiar to the Haredi population: “When I tell a story I will often change it. That is to say, I’ll make it more ‘Jewish.”’ Moreover, a few therapists also check the illustrations featured in the story books or in the games used in therapy and edit them to make them match the cultural symbols and modesty codes of Haredi society.

Some therapists pointed out that they very rarely use technological means such as smartphones or computers, and that they particularly avoid using recorded music. When they do use music, they make sure to use either instrumental tracks without lyrics or songs popular in the Haredi community. In rare instances, a few therapists feel that a certain song from their world can be of help to a client, and in that case they will only use the song if the lyrics bear no conflict with the norms of Haredi society and only after receiving direct approval from the client’s parents. Furthermore, a few therapists stated that they only sing with young children and refrain from singing with older boys or in the presence of parents, due to the Halachic ordinance which prohibits men from listening to women singing.

Similarly, a few therapists avoid using artistic materials such as paintings, magazines or miniatures that feature elements which Haredi society finds unacceptable. Sometimes a few therapists would hide props or materials through which children might express less socially-accepted content, such as aggression: “There is a lot of aggressive content that the kids want to engage with during therapy and they can’t because I make sure hide that kind of stuff in the room in advance.” It is interesting to note that when the therapists make the arts accessible by using terminology that is customary in Haredi society, even things that are problematic in terms of the Halacha, such as making statues or masks, can take place during therapy without unease or apprehension: “I’ve changed the terminology. The statues are called dolls, the totems are models or structures, the masks are called funny faces.”

Some therapists shared that they make these adjustments out of the comprehension that if they should allow the children to engage with things that are forbidden at home, they may provoke a resistance to therapy, ambivalence or confusion: “I didn’t bring any things that might destabilize or provoke any kind of ambivalence on the part of the client in an interventionist manner.”

### Systemic Aspects

#### The Relationship With the Parents Develops Gradually

When a Haredi child undertakes arts therapy, there is continuous contact with the client’s parents, including meeting at the beginning and end of the therapy, ongoing parental guidance, as well as occasional chats. The statements of most therapists revealed that the process of establishing a relationship with the parents takes place in stages. The stage of initial contact is often a problematic one because the parents are apprehensive about the therapist as an outsider to Haredi society. The cultural disparity makes the relationship with most parents cold, pragmatic and official. Sometimes it even leads to cessation of therapy: “A lot of treatments have ended because of suspicion, or didn’t take place because of suspicion… They don’t want the child to be in therapy alone with you.” Some therapists revealed that parents scrutinize the therapist thoroughly in the first stages of contact out of the desire to protect the children and their upbringing, for fear that the therapist might legitimize things that are not suitable to the lifestyle of the Haredi community: “They are automatically suspicious of me… That I’ll be some kind of figure who gives legitimacy to straying off the straight and narrow.” As a result, some therapists are forced to make an extra effort to prove their professionalism and sensitivity: “You need to prove yourself, both that you’re looking out for them and that you’re worth something, to get them on your side.” In order to gain the parents’ trust and establish a relationship with them, a few of the therapists encourage frank and direct discussions of the cultural difference: “I very much encourage talking about our differences… After we talk about it, it gets better.”

According to some therapists, once the parents learn to trust the therapist, they often express gratitude and appreciation. At this stage, the parents transition from suspicion to trust and dependency. They efface themselves before the therapist, whom they now perceive as a figure of authority: “The moment they begin to trust you, it is full on trust on the most absolute level. Any word I say is almost sacred.” Accordingly, the parents wish for the therapists to make decisions for them on various issues and have a hard time participating in joint deliberations: “They want me to decide… God says, the rabbi says, the principal says, and now the therapist will say.” It is important to note, however, that more often than not the rabbi’s opinion still remains the most influential factor in the parents’ decision-making process.

In most cases, establishing a relationship allows for a more open dialog about a variety of subjects. However, it would seem that when it comes to parents from certain ultra-Orthodox factions, there are still certain topics and issues that the therapist cannot address freely even when the relationship is relatively well-established: “It really depends on the faction… There were some with whom I had a very open dialog, but enough others where there was nothing I could do.” In most instances where trust has been established, the parents are able to commit and dedicate themselves to the therapy, even when it is long and intensive, and the therapists can see that they apply the ideas emerging from the sessions: “Parents commit themselves… Some parents say to me ‘now it’s something that belongs to me and my child’… There are even parents who do it with their other children.”

#### The Role of the Father in Therapy Is Variable

The therapists’ statements indicate that the faction to which the parents belong has a great effect on the role the father plays in the therapy. Thus, for example, while some therapists shared that they see fathers rarely and even when they are present, they do not make eye contact with the therapist and project a sense of discomfort, other cases had both parents in attendance: “I’ve had many families where only the mom ever came in. There were also those cases where the father would be in the room but not look at me. But there were also cases where both parents showed up.” It is interesting to note that in certain factions the situation is the exact opposite: the father plays a significant role in the child’s therapy, he dominates the discussion, makes eye contact and exhibits more responsibility and commitment than the mother.

#### Therapy for the Child Paves the Way for Therapy for the Parent

Another unique point that emerged from the statements of some therapists is that parents will frequently continue therapy with the therapist who treated their child, after the child’s therapy has concluded. In some cases, during the child’s therapy course, the therapist will recognize a need for one of the parents to get therapy and makes the suggestion to the parent. In other cases it is the parent who recognizes, at some point in their child’s process, that they themselves require therapy and asks the therapist to provide it: “We get half way through the therapy and the parents see a real change for the better in their child, so the dad says ‘I’m going to start therapy myself.” A few therapists remarked that often it is easier for the parent to send the child to therapy rather than invest family resources in themselves, and therefore sending the child to therapy is perceived by the therapist as a cry for help on the part of the parent: “A lot of times they bring the child to therapy but it’s the mother who’s crying out for help… And it’s happened more than once or twice that when the kid goes out, the mother goes in.” Nevertheless, a few therapists noted that there are many parents who are unwilling to look at themselves and realize that they are the ones who need help.

#### Characteristics of Work With the Parents

Some therapists maintained that the work they do with the parents includes teaching them what therapy is: “I am constantly trying to make therapy accessible to them.” For instance, they have to explain to the parents that therapy is a gradual process that may take a long time, and describe in detail how they work and the techniques involved.

Another aspect of working with the parents pertains to cultivating their ability to observe the child. Some therapists explained that since many parents are primarily focused on the technical day-to-day functioning rather than the emotional state of their child, they have to teach the parents to observe the child’s distress and to help them understand what causes it, what makes it more frequent or acute, and how it relates to the processes they are undergoing. Some therapists related that a central part of their work revolves around getting parents to understand that behavior is an expression of mental states. There is often a need to communicate the client’s emotional needs to the parents, help them recognize these needs as normal even in cases where they differ from their own personal needs or diverge from the norms of Haredi society, and together figure out how they can satisfy these needs and thereby alleviate the child’s emotional distress: “The most important thing is to show them how every external act is an expression of something happening in the psyche… To understand that the child is different from them… and to show what can be done.” A few therapists talked about teaching the parents to observe the child’s uniqueness and strengths, and working with them on their ability to admire their child and to show their admiration explicitly and emphatically: “That feeling of enthusiasm about the child, it’s not always put into words, and that’s something I often find myself working on with them.”

Some therapists added that they focus on the parents’ participation as partners in therapy. They expounded that even though they often respond to the parents’ requests for guidelines and practical tools, they invest most of their resources in encouraging joint deliberation: “They want me to decide, me to tell them… So I say – let’s see together, it’s not my decision… I’m here to deliberate with you.” A few of the therapists make sure the parents understand the importance of joint guidance sessions, which sometimes come at the expense of sessions with the child, while emphasizing the significance of their cooperation for the therapy’s success: “I always tell them: ‘Without you, it’s impossible.”’

#### Objectives in Working With Educational Staff and Spiritual Figures

Some therapists spoke about their relationships with educational staff and spiritual figures, such as teachers, rabbis and community leaders. According to some therapists, for the most part they maintain good working relationships with these figures, albeit not close or personal ones. The relationships are often closer with female figures, and less close with rabbis, however, it appears that these therapists consistently view this matter as largely dependent on the individual personality of each such figure. Sometimes they engage in direct dialog with the educational staff and spiritual figures; other times, however, the dialog take place through the mediation of the parents, which is the case when certain rabbis refuse to speak to the therapist directly due to her being a woman. Furthermore, sometimes a few therapists experience difficulties in getting in touch with these figures due to technical issues such as lack of telephone access.

It appears that work with these figures revolves around three main objectives. As stated previously, the first objective mentioned by some of the therapists is consulting with them in complex cases or when they encounter problems in the course of therapy, in order to learn about certain Halachic or cultural aspects and understand how they must behave. Some therapists emphasized the important of having a consulting agent within the Haredi community: “It’s very important to have guidance about cultural sensitivity… to have someone who knows the population instruct you.” Another objective that emerged out of the statements of a few therapists is to obtain information, for example about the child’s family background and their functioning in different areas of life: “It’s not just about seeing the child from another angle, but also about understanding how they are seen.” The third objective described by some therapists is to provide information. Some therapists stated that educational staff and spiritual figures sometimes consult with them when they need to make decisions pertaining to complex issues, and they are then sometimes required to explain concepts from their own foreign world to the Haredi society. A few therapists create a discourse focused on the client’s capacities and intended to communicate the mental motivations driving their behaviors, as well as instruct the interlocutors about the possible actions they can undertake in response to these behaviors.

## Discussion

The present study examined the perceptions of non-Haredi arts therapists regarding work with children from the Haredi community, thereby joining a previous study that examined the perceptions of non-Haredi dance/movement therapists working with Haredi children (Suskin and Karnieli, 2015) as well as other studies which examined psychotherapy (e.g., Freund and Band-Winterstein, 2017; Hess, 2018) and art therapy (Padolski-Kroper and Goldner, 2020) in adults from the Haredi community.

The study offers a glimpse into the encounter between fundamentally different cultures and value systems – the traditionalist-collectivist culture of the clients and their parents, on the one hand, and the Western culture that forms the basis of psychotherapy and to which the non-Haredi therapists belong, on the other (Schwartz, 2017; Gopalkrishnan, 2018). The study found significant disparities between therapists and clients in terms of perceptions and values. These include the disparity in self-perception that emerged in the study and is also described in the literature (Yafeh, 2001; Schlesinger and Russo-Netzer, 2017), disparities in family structures and the number of children in the family, and the therapists’ perception of various behaviors in some children (e.g., difficulty making choices, a desire to please and overall restraint) as focal points for therapeutic work as opposed to the perception described in the literature of these behaviors as conforming to the values and norms of ultra-Orthodox society (Goodman, 2003; Frosh, 2004; Hakak, 2011). The findings indicate that these disparities are fundamental to the experience of all parties involved in the therapy (therapists, parents and children) and present them with extremely complex challenges. Thus, the present study is an addition to the body of research dealing with the complexity typical of cross-cultural therapy (e.g., Qureshi and Collazos, 2011).

With regard to parents, it would seem that these disparities cause them apprehensions which often express themselves in difficulties trusting the therapist, the tendency to over-scrutinize her, and sometimes even a cessation of therapy due to the cultural difference. The reason for this seems to be rooted in the fear that values opposed to those of the Haredi worldview might penetrate through to the child (Band-Winterstein and Freund, 2015), as well as the parents’ dread of potential harm to their child’s spiritual upbringing (Schnitzer et al., 2011). Similarly, the children also sometimes struggle to trust and bond with the therapist, and they too find themselves preoccupied with the disparities that sometimes charge the therapeutic relationship with tension. The interviews indicate that the therapists choose to accord a central place to the values and norms of the cultural world of the child and their parents, even at the cost of restrictions and strains on their experience. Thus, for example, they sometimes feel limited in terms of the content that may be discussed in therapy and the artistic tools they can use, they find it hard to be authentic, and in many cases they find themselves adapting to societal norms that are not part of their own sets of values. These findings are consistent with previous research that also described the difficulties experienced by non-Haredi therapists due to the need to be considerate of Haredi culture and values, and to some extent compromise their professional outlook and worldviews (Suskin and Karnieli, 2015). Moreover, the findings of the present study show that the challenges faced by therapists due to cultural disparities amount not only to setting their own values aside, but sometimes to actual distress and harm, as reflected in their descriptions of dealing with feelings of intrusion and invasion of privacy. The therapists also described dealing with situations of conflicts of values, on the subject of nationalist ideology for instance, which in some cases caused them distress. It seems that despite their sincere attempts to conduct therapy in accordance with the cultural competence approach (Qureshi and Collazos, 2011; Sue and Sue, 2013), the therapists encounter inevitable failures.

Nonetheless, it is imperative to note that the therapists also mentioned advantages directly linked to the cultural difference between themselves and their clients. These include the opportunity for candor that is often made possible because the therapist does not belong to the Haredi community and therefore cannot lead to the exposure of secrets, as well as the sense of control that children sometimes experience due to their ability to teach the therapist about their world. The therapist too may benefit from the cultural gap in terms of personal growth, including introspection and the development of capacities such as humility and patience. These findings are in line with previous descriptions of cross-cultural therapies in the literature, suggesting that alongside challenges and difficulties, these encounters may strengthen the client’s sense of security and thereby increase engagement and facilitate candor (Stolovy et al., 2012), enhance parents’ ability to maintain a therapeutic relationship (Snir et al., 2017), and promote personal and professional growth in therapists (Kapitan, 2015; Lee, 2017).

The findings point to areas of struggle and challenge that are unique to arts therapy with ultra-Orthodox children, including different forms of symbolic play and release capabilities compared to secular children, which may be expressed as a tendency to stick to the boundaries of concrete reality, restrained and stilted conduct, and focus on precision and aesthetics in their creative work. A similar picture, albeit with respect to older female clients from the Haredi population, emerged from a previous study which found that these clients had difficulty entering a playful space, feared uncontrolled exposure, and displayed limited ability to create expressive art (Padolski-Kroper and Goldner, 2020). Furthermore, the findings shed light on additional challenging areas such as the challenge of dealing with the body and the need to adapt the artistic activities to the laws and customs of Haredi society. At the same time, the therapists who participated in the study sought to emphasize that in most cases the children are able to express emotional experiences and engage with content and issues that are meaningful to them. According to the therapists, the main advantage of administering arts therapy to children from the ultra-Orthodox community lies in the distancing made possible by the artistic activities. As argued by the literature, since the art is separate from the creator, it renders the threatening content external to them (Schaverien, 1994) and thereby allows the client to engage with content that they would not have engaged with directly with the therapist (Lewis, 1992).

The study’s findings point to a multifaceted process of change taking place in Haredi society in relation to therapy in general, and to arts therapy in particular. Similarly to previous studies that have pointed out the complexity involved in this process of change (e.g., Freund and Band-Winterstein, 2013), the present study suggests that, as implied by the very nature of processes in general, shifts in attitudes can be identified alongside the perseverance of past perceptions regarding therapy. For example, the therapists who participated in the study did point to an improvement in the perception of the therapy as positive and beneficial by parents, community rabbis and members of the educational staff, which is also reflected in the fact that the clients’ parents sometimes turn to therapy themselves, following the child’s experience. even though, according to the therapists, some of them still do not quite grasp the full meaning of therapy or perceive it as a tool for fixing the child’s and the family’s problems. The therapists talked about parents who commit themselves to the therapy and implement its emergent conclusions; at the same time, similar to educational staff and spiritual figures, some of them tend to focus more on the effective benefits of therapy, expect quick results and seem to have difficulty perceiving therapy as a process. Nevertheless, it should be noted that the expectation of quick results may stem from the socio-economic characteristics of Haredi society, such as the large number of children in each family, which leads to high parental preoccupation, as well as multiple expenditures in conjunction with low income and high poverty rates (Cahaner and Malach, 2019). Thus, it can be assumed that reality floods parents with an aspiration for therapy to be as quick, efficient and cost-effective as possible. Similarly, it is likely that difficulties in perceiving therapy as a process stems primarily from a lack of familiarity with psychotherapy. Moreover, alongside the therapists’ descriptions of incidents in which children and parents share content that is not acceptable in Haredi society, there are reports of a general sense of secrecy and difficulties in revealing things, probably out of the fear of stigma, which often engenders an aversion to exposing problems or deficiencies (Greenberg et al., 2012; Barth and Ben-Ari, 2014). The therapists also noted that sometimes, in particularly sensitive and complex cases, the therapy is stopped prematurely and the client is transferred to care within the ultra-Orthodox community. This is in line with the preference for receiving therapy from professionals belonging to Haredi society that appears in the literature (Mozes et al., 2010; Schlesinger and Russo-Netzer, 2017).

These mixed attitudes, which may indicate a multifaceted process amounting to gradual change, are also reflected in the parents’ relationship with the therapist. In many instances, the process of establishing a relationship with the parents is challenging, and accompanied by various apprehensions and careful scrutiny of the therapist by the parents. However, in cases where the therapist manages to form a relationship, most parents perceive her as a professional and an authority figure, and show her respect and appreciation. According to the therapists, the perception of the therapist as an authority figure can sometimes lead parents to become dependent on her for decision-making regarding their child’s upbringing, and this sometimes makes it difficult for them to act as partners in the therapy and engaging in joint deliberations. This finding is explained in the literature, which describes how the entrenched norm of subordination to authority in Haredi society leads to a projection of responsibility on the therapist based on her perception as an authority figure. But while the references to this issue in the literature pertain to cases where instructions given by therapists have the potential to provoke conflict (Hess, 2018), the present study expands and clarifies that dependence on the therapist may be broader and deeper in the case of child therapy. Once the relationship with the parents is established, it often leads to a more open dialog; however, in some cases, there are still issues that remain excluded from discussion, such as content that the parents are not willing for the child to engage with due to cultural or religious reasons, as well as any issues they may have with the therapist herself. These findings appear to be consistent with the literature, which claims that the restrictions on lifestyle in ultra-Orthodox society delimit the therapeutic discourse within the boundaries of the permissible and the forbidden according to religious laws and cultural norms (Hess and Pitariu, 2011).

The present study sought to examine arts therapy with Haredi children from the perspective of therapists outside of this society. It is important to note that the study is based on interviews with arts therapists and therefore reflects their subjective perceptions. Examining cross-cultural arts therapy in children from ultra-Orthodox communities through the perceptions of the other partners in the therapy, including the children themselves, the parents, as well as educational staff and spiritual figures, would enrich this perspective and help cross-validate the findings. It would also be worthwhile to conduct a comparative examination of the therapists’ perspective and the Haredi point of view in a follow-up study. Moreover, all the participants in the present study were women. Future research should focus on a comparative examination of the experiences and perceptions of women therapists and male therapists to delve deeper into the possible effects of gender on therapy. Future studies could also shed more light on potential differences between modalities of arts therapies in therapy for Haredi children. Likewise, further research that involves gathering quantitative information through an attitude survey based on the perceptions and experiences of the therapists described in the present study, may provide an overview of the prevalence of the experiences described in the study and help assess development and change over time.

## Data Availability Statement

The datasets for this manuscript are not publicly available to protect participants’ confidentiality. Requests to access the datasets should be directed to LK, lali.livyatan@gmail.com.

## Ethics Statement

The studies involving human participants were reviewed and approved by Ethics committee for the Faculty of Social Welfare and Health Sciences at the University of Haifa. The patients/participants provided their written informed consent to participate in this study. Written informed consent was obtained from the individual(s) for the publication of any potentially identifiable images or data included in this article.

## Author Contributions

This research is part of a Ph.D. dissertation, which was initiated, planned, coordinated, and conducted by LK. She collected the data, carried out the analyses, and wrote the manuscript. DR and SS supervised the Ph.D. dissertation and assisted in all stages. All authors contributed to the article and approved the submitted version.

## Conflict of Interest

The authors declare that the research was conducted in the absence of any commercial or financial relationships that could be construed as a potential conflict of interest.
