# Supplementary material for: Non-Haredi Arts Therapists’ Perceptions of Therapy With Ultra-Orthodox Children
Source: Front Psychol. 2021 Feb 4;12:599872. doi: 10.3389/fpsyg.2021.599872 (PMC7889799; doi:10.3389/fpsyg.2021.599872)
Supplement: Supplementary file 1 [file Table_1.docx]

Supplementary Material

# Supplementary Tables

## Table 1

Table 1. Therapists' Demographics

| **No.** | **Specialty** | **Age** | **Years of experience in treating Ultra-Orthodox children** | **Religious outlook** |
| --- | --- | --- | --- | --- |
| 1 | Dance | 31–40 | 1–5 | Secular |
| 2 | Dance | 31–40 | 1–5 | Secular |
| 3 | Dance | 31–40 | 6–10 | Secular |
| 4 | Dance | 31–40 | 1–5 | Secular |
| 5 | Dance | 51–60 | 6–10 | Secular |
| 6 | Dance | 51–60 | 16–20 | Secular |
| 7 | Bibliotherapy | 31–40 | 1–5 | Secular |
| 8 | Bibliotherapy | 31–40 | 1–5 | Secular |
| 9 | Bibliotherapy | 41–50 | 16–20 | Secular |
| 10 | Bibliotherapy | 51–60 | 1–5 | Secular |
| 11 | Visual arts | 31–40 | 6–10 | Religious Zionist |
| 12 | Visual arts | 51–60 | 11–15 | Secular |
| 13 | Visual arts | 61–70 | 21–25 | Secular |
| 14 | Psychodrama | 31–40 | 1–5 | Religious Zionist |
| 15 | Psychodrama | 41–50 | 1–5 | Religious Zionist |
| 16 | Music | 41–50 | 1–5 | Secular |
| 17 | Music | 51–60 | 26–30 | Secular |
